# MLatom 3: A Platform for Machine Learning-Enhanced Computational Chemistry Simulations and Workflows

**DOI:** 10.1021/acs.jctc.3c01203

**Published:** 2024-01-25

**Authors:** Pavlo O. Dral, Fuchun Ge, Yi-Fan Hou, Peikun Zheng, Yuxinxin Chen, Mario Barbatti, Olexandr Isayev, Cheng Wang, Bao-Xin Xue, Max Pinheiro Jr, Yuming Su, Yiheng Dai, Yangtao Chen, Lina Zhang, Shuang Zhang, Arif Ullah, Quanhao Zhang, Yanchi Ou

**Affiliations:** † State Key Laboratory of Physical Chemistry of Solid Surfaces, College of Chemistry and Chemical Engineering, and Innovation Laboratory for Sciences and Technologies of Energy Materials of Fujian Province (IKKEM), Xiamen University, Xiamen, Fujian 361005, China; ‡ Fujian Provincial Key Laboratory of Theoretical and Computational Chemistry, Xiamen, Fujian 361005, China; § Aix Marseille University, CNRS, ICR, Marseille 13013, France; ∥ Institut Universitaire de France, Paris 75231, France; ⊥ Department of Chemistry, Carnegie Mellon University, Pittsburgh, Pennsylvania15213, United States; # iChem, Xiamen University, Xiamen, Fujian 361005, China; ∇ School of Physics and Optoelectronic Engineering, Anhui University, Hefei230601, China

## Abstract

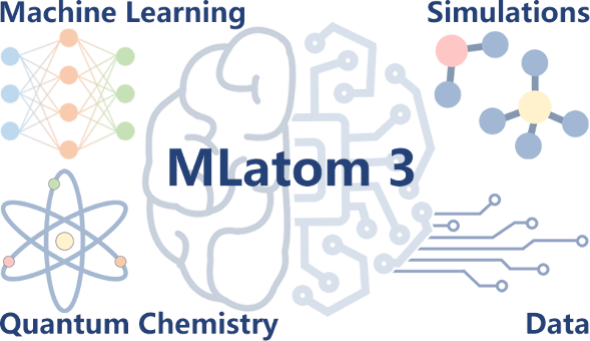

Machine learning (ML) is increasingly becoming a common tool in computational chemistry. At the same time, the rapid development of ML methods requires a flexible software framework for designing custom workflows. MLatom 3 is a program package designed to leverage the power of ML to enhance typical computational chemistry simulations and to create complex workflows. This open-source package provides plenty of choice to the users who can run simulations with the command-line options, input files, or with scripts using MLatom as a Python package, both on their computers and on the online XACS cloud computing service at XACScloud.com. Computational chemists can calculate energies and thermochemical properties, optimize geometries, run molecular and quantum dynamics, and simulate (ro)vibrational, one-photon UV/vis absorption, and two-photon absorption spectra with ML, quantum mechanical, and combined models. The users can choose from an extensive library of methods containing pretrained ML models and quantum mechanical approximations such as AIQM1 approaching coupled-cluster accuracy. The developers can build their own models using various ML algorithms. The great flexibility of MLatom is largely due to the extensive use of the interfaces to many state-of-the-art software packages and libraries.

## 1 Introduction

Computational chemistry simulations are common in chemistry research thanks to abundant general-purpose software, most of which have started as purely quantum mechanical (QM) and molecular mechanical (MM) packages. More recently, the rise of artificial intelligence (AI)/machine learning (ML) applications for chemical simulations has caused the proliferation of programs mostly focusing on specific ML tasks such as learning potential energy surfaces (PESs).^1−17^ The rift between the development of the traditional QM and MM packages on the one hand and ML programs on the other hand is bridged to some extent by the higher-level library ASE,^18^ which enables usual computational tasks via interfacing heterogeneous software. The further integration of QM, MM, and ML has been prompted by the maturing of ML techniques and is evidenced by the growing trend of incorporating ML methods in the QM and MM computational chemistry software.^13,19−21^

Against this backdrop, the MLatom package started in 2013 as a pure standalone ML package to provide a general-purpose experience for computational chemists akin to the black-box QM packages.^22^ The early MLatom could be used for training, testing, and using ML models and their combinations with QM methods (e.g., Δ-learning^23^ and learning of Hamiltonian parameters^24^), accurate representation of PES,^25,26^ sampling of points from data sets,^26^ ML-accelerated nonadiabatic dynamics,^27^ and materials design.^28^ The fast pace of method and software development in QM, MM, ML, and other computational science domains led to MLatom 2, which started to include interfaces to third-party packages.^29^ Such an approach provided a unique opportunity for the package users to choose one of the many established ML models, similar to the users of the traditional QM software who can choose one of the many QM methods. MLatom 2 could perform training of the ML models, evaluate their accuracy, and then use the models for geometry optimization and frequency calculations. Special workflows were also implemented, such as acceleration of the absorption UV/vis spectra calculations with ML^30^ and prediction of two-photon absorption spectra.^31^ In addition, MLatom 2 could be used to perform simulations with the general-purpose AI-enhanced QM method^32^ AIQM1 and universal machine learning potentials of the ANI family^2,33−35^ with the accurate scheme developed for calculating heats of formation^36^ with uncertainty quantification with these methods.

With time, the need to develop increasingly complex workflows that incorporate ML and QM for a broad range of applications has necessitated the rethink and redesign of MLatom to enable the rapid development of highly customized routines. These additional design requirements for MLatom to serve not only as a black-box general-purpose package but also as a flexible platform for developers resulted in a significant extension, redesign, and rewrite of the program. The subsequent upgrade has allowed the use of MLatom through the versatile Python API (MLatom PyAPI) and also included the implementation of more simulation tasks, such as molecular and quantum dynamics, and the support of QM methods and composite schemes based on the combinations of QM and ML models. This upgrade was released^37^ as MLatom 3 in 2023, 10 years after the start of the project. During this decade, MLatom went through a drastic transformation from a pure Fortran package to a predominantly Python package, with one-third of the code written in Fortran for efficient implementations of critical parts. MLatom 3 comes under the open-source permissive MIT license (modified to request proper citations), and the source code is available on open repositories so that, e.g., external developers are encouraged to contribute to the main project and may create their independent, derived, projects. Here, we give an overview of the capabilities of MLatom 3 and provide examples of its applications.

## 2 Overview

**Figure 1:**
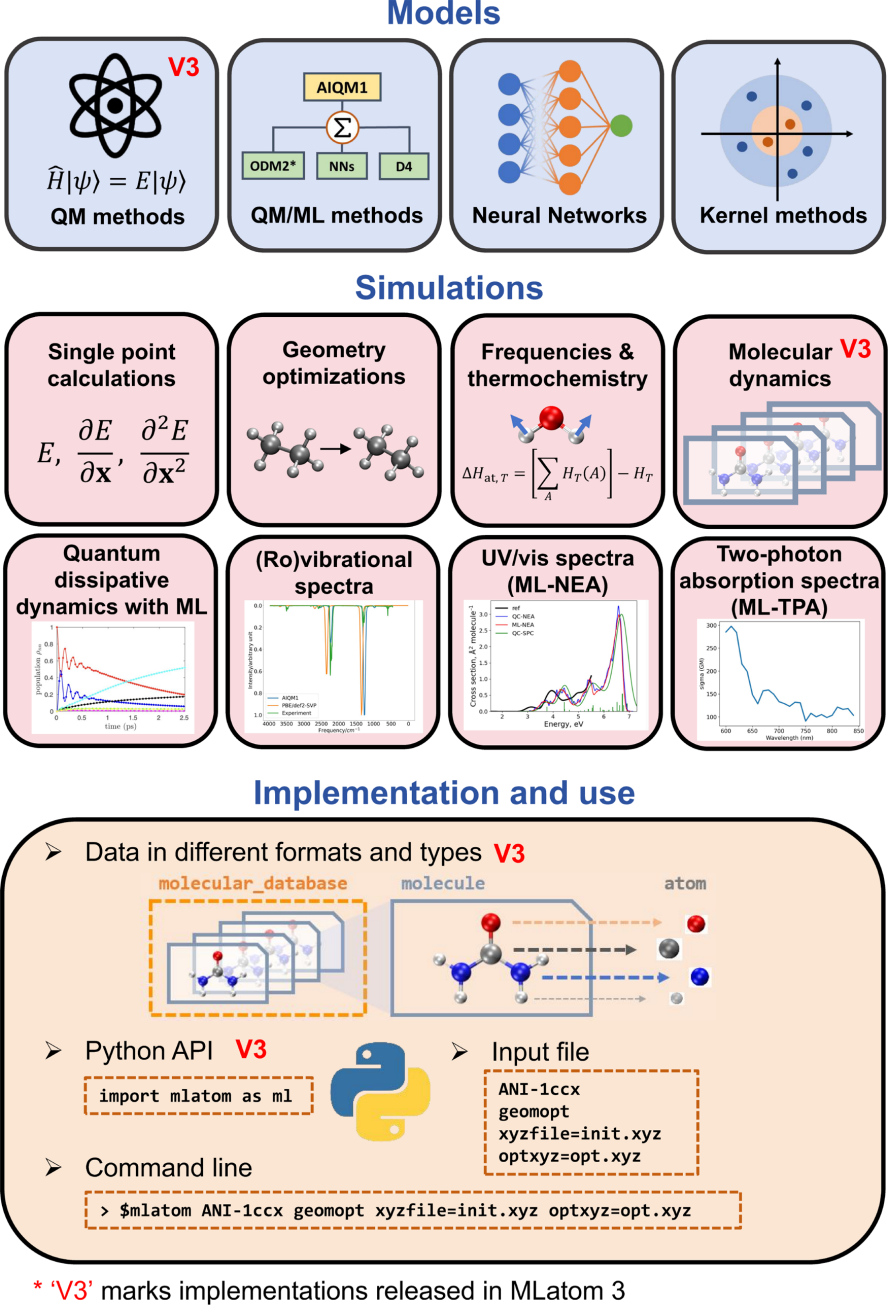
Overview of MLatom 3 capabilities. The plot in panel “Quantum dissipative dynamics with ML” is adapted with permission from ref (38). Copyright 2022, the Authors. The plot in panel “UV/vis spectra (ML-NEA)” is adapted from ref (29). Copyright 2021, the Authors.

MLatom merges the functionality from typical quantum chemical and other atomistic simulation packages with the capabilities of desperate ML packages, with a strong focus on molecular systems. The user can choose from a selection of ready-to-use QM and ML models and design and train ML models to perform the required simulations. The bird’s view of the MLatom capabilities is best given in Figure 1.

One of the current main goals of MLatom is to enable simulation tasks of interest for computational chemists with generic types of models that can be based on ML, QM, and their combinations (see Section 4). These tasks include single-point calculations, optimization of geometries of minima and transition states (which can be followed by intrinsic reaction coordinate (IRC) analysis^39^), frequency and thermochemical property calculations, molecular and quantum dynamics, rovibrational (infrared (IR) and power) spectra, ML-accelerated UV/vis absorption, and two-photon absorption spectra simulations. This part of MLatom is more similar to traditional QM and MM packages but with much more flexibility in model choice and unique tasks. A dedicated Section 5 will give a more detailed account of the simulations.

Enabling the users to create their own ML models was MLatom’s original main focus, and it continues to play a major role. The MLatom supports a range of carefully selected representative ML algorithms that can learn the desired properties as a function of the 3D atomistic structure. Typically, these algorithms are used, but not limited to, for learning PESs and hence often can be called, for simplicity, ML (interatomic) potentials (MLPs).^40−44^ One particular specialization of MLatom is the original implementation of kernel ridge regression (KRR) algorithms for learning any property as a function of any user-provided input vectors or *XYZ* molecular coordinates.^22^ In addition, the user can create custom multicomponent models based on concepts of Δ-learning,^23^ hierarchical ML,^25^ and self-correction.^26^ These models may consist of the ML and QM methods. MLatom provides standardized means for training, hyperparameter optimization, and evaluation of the models so that switching from one model type to another may need just one keyword change.^29^ This allows one to easily experiment with different models and choose the most appropriate one for the task.

The data are as important as choosing and training the ML algorithms. MLatom 3 provides several data structures specialized for computational chemistry needs, mainly based on versatile Python classes for atoms, molecules, molecular databases, and dynamics trajectories. These classes allow not just storing the data in a clearly structured format but also handling it by, e.g., converting to different molecular representations and data formats and splitting and sampling the data sets into the training, validation, and test subsets. Because data structure is a central concept in the age of data-driven models and MLatom as a package, we describe data structures in Section 3 before describing models, simulations, and machine learning.

**Figure 2:**
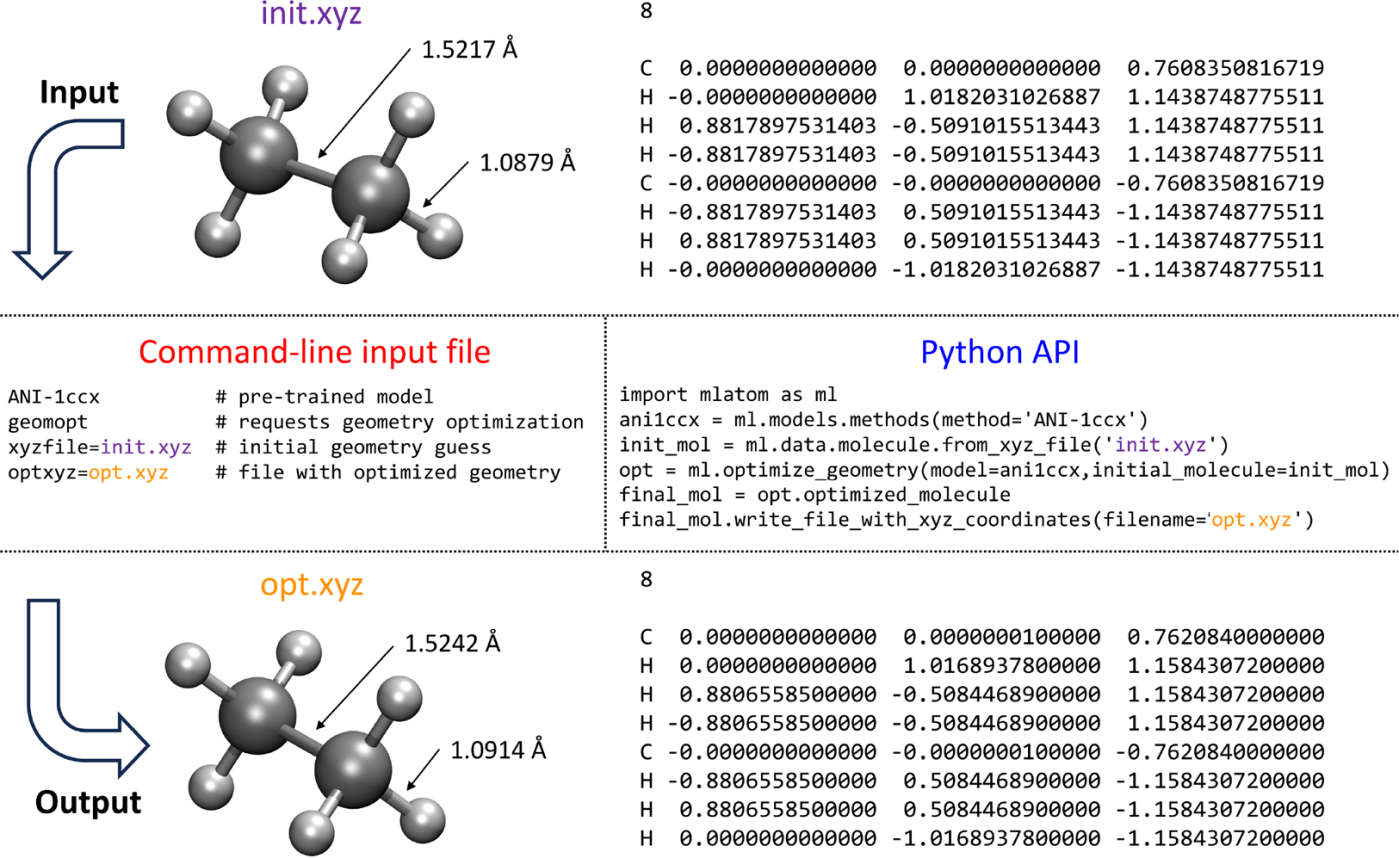
Side-by-side comparison of the usage of MLatom in both the command-line mode and via Python API for a common task of geometry optimization with one of the pretrained ML models ANI-1ccx.

How the user interacts with the program is also important, and ideally, the features should be easily accessible and their use intuitive. MLatom calculations can be requested by providing command-line options either directly or through the input file. Alternatively, MLatom can be used as a Python module, which can be imported and used for creating calculation workflows of varying complexity. A side-by-side comparison of these two approaches is given in Figure 2. More examples highlighting different use cases of MLatom are interspersed throughout this article.

MLatom as an open-source package can be conveniently installed via PyPI, i.e., simply using the command pip install mlatom or from the source code available on GitHub at https://github.com/dralgroup/mlatom. To additionally facilitate access to AI-enhanced computational chemistry, MLatom can be conveniently used in the XACS cloud computing service at https://XACScloud.com whose basic functionality is free for noncommercial uses such as education and research. Cloud computing eliminates the need for program installation and might be particularly useful for users with limited computational resources.

## 3 Data

**Figure 3:**
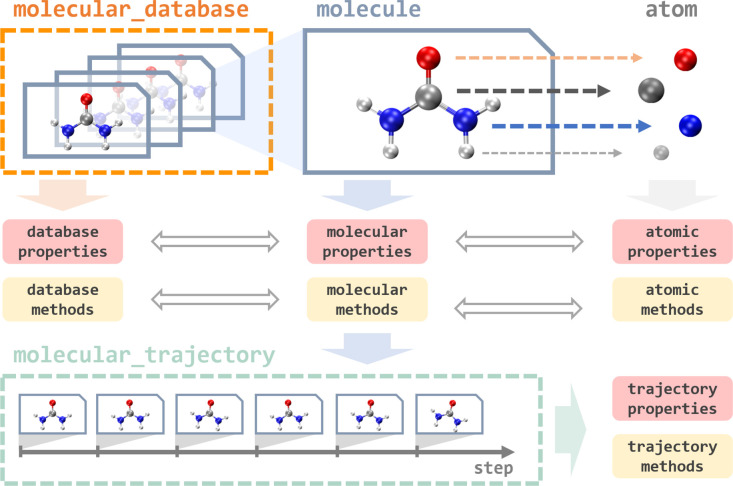
Overview of different data types in MLatom.

In MLatom, everything revolves around operations on data: databases and data points of different types, such as an atom, molecule, molecular database, and molecular trajectory (Figure 3). They are implemented as Python classes that contain many useful properties and provide different tools to load and dump these data-type objects using different formats. For example, the key type is a molecule that can be loaded from an *XYZ* file or SMILES and then automatically parsed into the constituent atom objects. Atom objects contain information about the nuclear charge and mass as well as nuclear coordinates. A molecule object is assigned with charge and multiplicity. Information about molecular and atomic properties can be passed to perform simulations, e.g., MD, with models that update and create new molecule objects with calculated quantum mechanical properties such as energies and energy gradients.

See Figure 2 for an example of loading a molecule object init_mol from the file init.xyz, used as the initial guess for the geometry optimization, returning an optimized geometry as a new molecule object final_mol, which is saved into the opt.xyz file. Data objects can be directly accessed and manipulated via the MLatom Python API. When using the MLatom in the command-line mode, many similar operations are done under the hood so that the user often just needs to prepare input files in standard formats such as files with *XYZ* coordinates.

Molecule objects can be combined into or created by parsing the molecular database that has functions to split it into the different subsets needed for training and validation of ML models. The databases can be loaded and dumped in plain text (i.e., several files including *XYZ* coordinates, labels, and *XYZ* derivatives), JSON, and npz formats. Another data type is molecular trajectory, which consists of steps containing molecules and other information. Molecular trajectory objects are created during geometry optimization and MD simulations, and in the latter case, the step is a snapshot of MD trajectory, containing information about the time, nuclear coordinates and velocities, atomic numbers and masses, energy gradients, kinetic, potential, and total energies, and, if available, dipole moments and other properties. The trajectories can be loaded and dumped in JSON, H5MD,^45^ and plain text formats.

Molecules for which *XYZ* coordinates are provided can be transformed in several supported descriptors: inverse internuclear distances and their version normalized relative to the equilibrium structure (RE),^26^ Coulomb matrix,^46,47^ and their variants.^29^

MLatom also has separate statistics routines to calculate different error measures and perform other data analyses.^29^ Routines for preparing common types of plots, such as scatter plots and spectra, are available too.

## 4 Models and Methods

**Figure 4:**
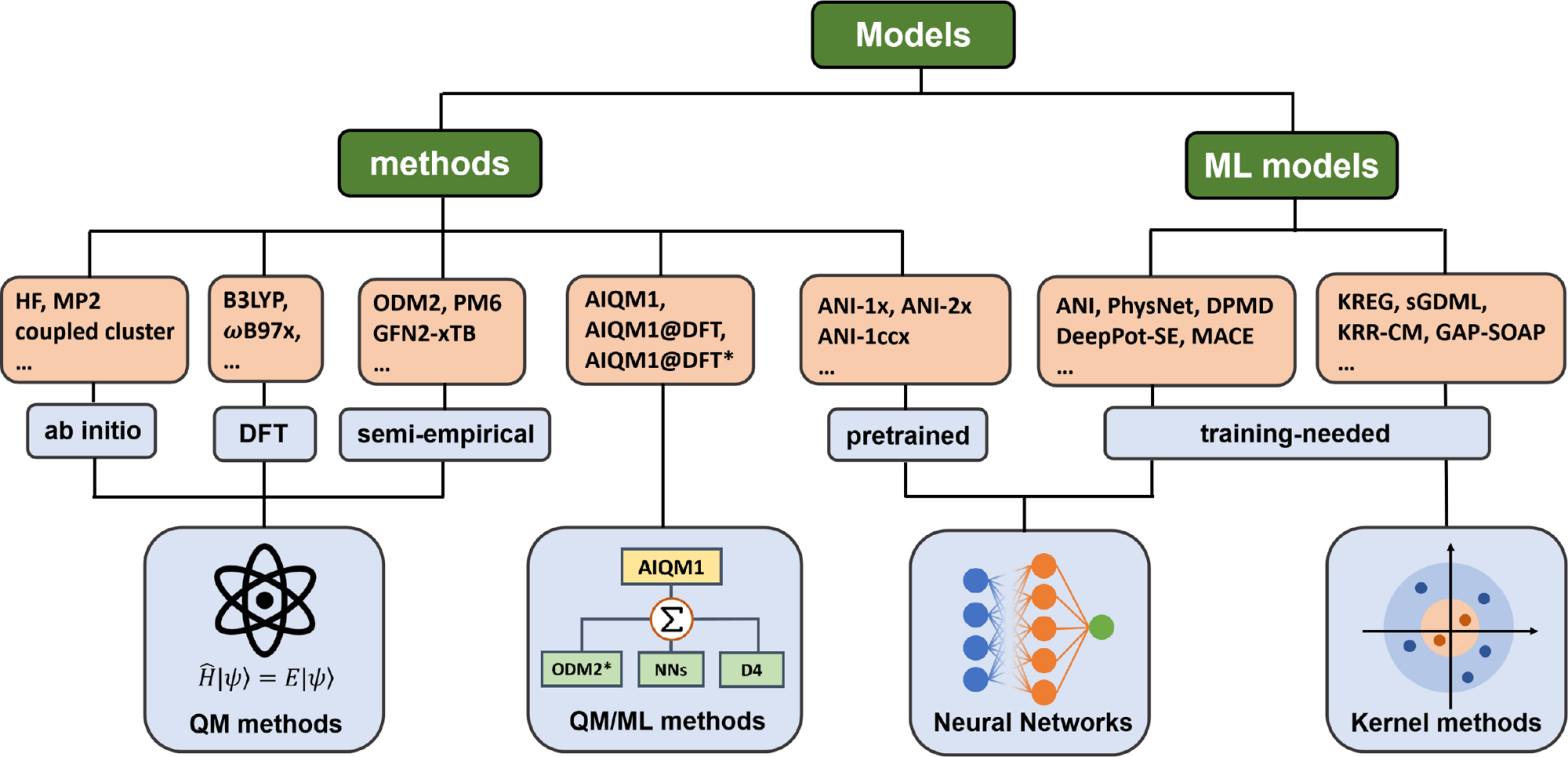
Overview of different model types in MLatom.

Any of the simulations need a model that provides the required output for a given input. The architecture and algorithms behind the models can be designed by an expert or chosen from the available selection. ML models typically require training to find their parameters before they can be used for simulations. Some of these models, such as universal MLPs of the ANI family,^2,33−35^ are already pretrained for the user who does not have to train them. This is similar to QM methods, which are commonly used out-of-the-box without tuning their parameters. In MLatom, we call a method any model that can be used out-of-the-box for simulations. Both pretrained ML models and QM methods belong to the methods in MLatom’s terminology, which is reflected in the keyword names. This model type also includes hybrid pretrained ML and QM methods. Below, we overview models available in MLatom when writing this article, the selection of available methods and models with provided architectures that need to be trained, and the ways to design custom models (Figure 4 and Table 1).

**Table 1:** Overview of Models in MLatom 3 and Their Implementations

model type	model name	implementation
Methods (models that can be used without training)		
QM methods	*ab initio* methods, DFT	interfaces to PySCF^48^, Gaussian^48^
	semiempirical OMx^49^, DFTB, NDDO-type methods	interfaces to MNDO^50^, Sparrow^51^
	semiempirical GFNx-TB^52^ methods	interface to xtb^53^
	CCSD(T)*/CBS^34^	interface to Orca^54,55^
QM/ML methods	AIQM1, AIQM1@DFT, AIQM1@DFT*^32^	interfaces to MNDO^50^ and Sparrow^51^ for the ODM2*^32,49^ part, TorchANI^2^ for the NN part, dftd4^56^ for D4 corrections^57^
pretrained ML models	ANI-1x^33^, ANI-2x^35^, ANI-1ccx^34^	interface to TorchANI^2^
Models needing training		
neural networks	MACE^58,59^	interface to MACE^60^
	ANI-type^2^	interface to TorchANI^2^
	DPMD^61^, DeepPot-SE^62^	interface to DeePMD-kit^63^
	PhysNet^64^	interface to PhysNet^64^
kernel methods	(p)KREG^26,65^	native implementation
	sGDML^66^	interface to sGDML^3^
	KRR-CM^46,47^	native implementation
	GAP^67^-SOAP^68^	interfaces to GAP suite^67^ and QUIP^69^

### 4.1 Methods

MLatom provides access to a broad range of methods through interfaces to many third-party, state-of-the-art software packages:

- Pretrained ML models:
  - Universal potentials ANI-1ccx,^34^ ANI-1x,^33^ ANI-2x,^35^ ANI-1x-D4, and ANI-2x-D4. ANI-1ccx is the most accurate and approaches gold-standard CCSD(T) accuracy. We have seen an example of its use in geometry optimization in Figure 2. Other methods approach the density functional theory (DFT) level. ANI-1ccx and ANI-1x are limited to CHNO elements, while ANI-2x can be used for CHNOFClS elements. We allow the user to use D4 dispersion-corrected universal ANI potentials that might be useful for noncovalent complexes. D4 correction^57^ is taken for the ωB97X functional^70^ used to generate data for pretraining ANI-1x and ANI-2x. ANI models are provided via an interface to TorchANI^2^ and D4 corrections via the interface to dftd4.^56^ These methods are limited to predicting energies and forces for neutral closed-shell compounds in their ground state. MLatom reports uncertainties for calculations with these methods based on the standard deviation between neural network (NN) predictions.^36^
  - The special ML-TPA model for predicting the two-photon absorption (TPA) cross sections.^31^
- Hybrid QM/ML methods AIQM1, AIQM1@DFT, and AIQM1@DFT*^32^ are more transferable and accurate than pretrained ML models but slower (the speed of semiempirical QM methods, which are still much faster than DFT). AIQM1 is approaching gold-standard CCSD(T) accuracy, while AIQM1@DFT and AIQM1@DFT* target the DFT accuracy for neutral, closed-shell molecules in their ground state. All these methods are limited to the CHNO elements. AIQM1 and AIQM1@DFT include explicit D4 dispersion corrections for the ωB97X functional, while AIQM1@DFT* does not. They also include modified ANI-type networks and the modified semiempirical QM method ODM2^49^ (ODM2*, provided by either the MNDO^50^ or Sparrow^51^ program). These methods can also be used to calculate charged species, radicals, excited states, and other QM properties such as dipole moments, charges, oscillator strengths, and nonadiabatic couplings. MLatom reports uncertainties for calculations with these methods based on the standard deviation between NN predictions.^36^
- A range of established QM methods from *ab initio* (e.g., HF, MP2, coupled cluster, *etc*.) to DFT (e.g., B3LYP,^71,72^ ωB97X,^70^*etc*.) via interfaces to PySCF^48^ and Gaussian.^48^
- A range of semiempirical QM methods (GFN2-xTB,^52^ OM2,^73^ ODM2,^49^ AM1,^74^ PM6,^75^*etc*.) via interfaces to the xtb,^53^ MNDO,^50^ and Sparrow^51^ programs.
- A special composite method CCSD(T)*/CBS^34^ extrapolating CCSD(T) to the complete basis set via an interface to Orca.^54,55^ This method is relatively fast and accurate. It allows the user to check the quality of calculations with other methods and generate robust reference data for ML. This method was used to generate the reference data for AIQM1 and ANI-1ccx.

### 4.2 Available Standard Models Needing Training

The field of MLPs is very rich in models. Hence, the user can often choose one of the popular MLP architectures reported in the literature rather than developing a new one. MLatom provides a toolset of MLPs from different types (see ref (40) for an overview and ref (29) for implementation details). These supported types can be categorized in a simplified scheme as follows:

- Models based on neural networks (NNs) with fixed local descriptors to which ANI-type MLPs^2^ and DPMD^61^ belong and with learned local descriptors represented by PhysNet^64^ and DeepPot-SE.^62^ MLatom also supports a representative equivariant NN MACE, which shows superior performance for many tasks.^58,59^
- Models based on kernel methods (KMs)^76^ with global descriptors to which (p)KREG,^26,65^ sGDML,^66^ and KRR-CM^46,47^ belong as well as with local descriptors represented by only GAP^67^-SOAP.^68^

Any of these models can be trained and used for simulations, e.g., geometry optimizations or dynamics. MLatom also supports hyperparameter optimization with many algorithms including grid search,^22^ Bayesian optimization via the hyperopt package,^77,78^ and standard optimization algorithms available in SciPy.^79^ Generalization errors of the resulting models can also be evaluated in standard ways (hold-out and cross-validation). More on this is available in a dedicated Section 6.

### 4.3 Custom Models Based on Kernel Methods

MLatom also provides the flexibility of training custom models based on kernel ridge regression (KRR) for a given set of input vectors **x** or *XYZ* coordinates and any labels **y**.^80,81^ If *XYZ* coordinates are provided, they can be transformed in one of the several supported descriptors (e.g., inverse internuclear distances and their version normalized relative to the equilibrium structure (RE) and the Coulomb matrix). The user can choose from one of the implemented kernel functions, including the linear,^22,81,82^ Gaussian,^22,81,82^ exponential,^22,81,82^ Laplacian,^22,81,82^ and Matérn^22,81−83^ as well as periodic^82,84,85^ and decaying periodic^82,84,86^ functions, which are summarized in Table 2. These kernel functions *k*(**x**, **x**_*j*_; **h**) are key components required to solve the KRR problem of finding the regression coefficients α of the approximating function *f̂*(**x**; **h**) of the input vector **x**:^80,81^
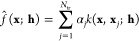
1

**Table 2:** Summary of the Available Kernel Functions for Solving the Kernel Ridge Regression Problem (Eq. 1) as Implemented in MLatom.

Kernel function	Formula	Hyperparameters in kernel function
Linear	*k*(**x**, **x**_*j*_) = **x**^T^**x**_*j*_	
Gaussian		σ > 0, length scale
exponential		σ > 0, length scale
Laplacian		σ > 0, length scale
Matérn		σ > 0, length scale; *n* is a non-negative integer
periodic		σ > 0, length scale; *p* > 0, period
decaying periodic		σ > 0, length scale; *p* > 0, period; *σ_p_* > 0, length scale for the periodic term

The kernel function, in most cases, has hyperparameters **h** to tune, and they can be viewed as measuring similarity between the input vector **x** and all of the *N*_tr_ training points **x**_*j*_ (both vectors should be of the same length *N*_*x*_). In addition to the hyperparameters in the kernel function, all KRR models have at least one more regularization parameter, λ, used during training to improve the generalizability.

### 4.4 Composite Models

**Figure 5:**
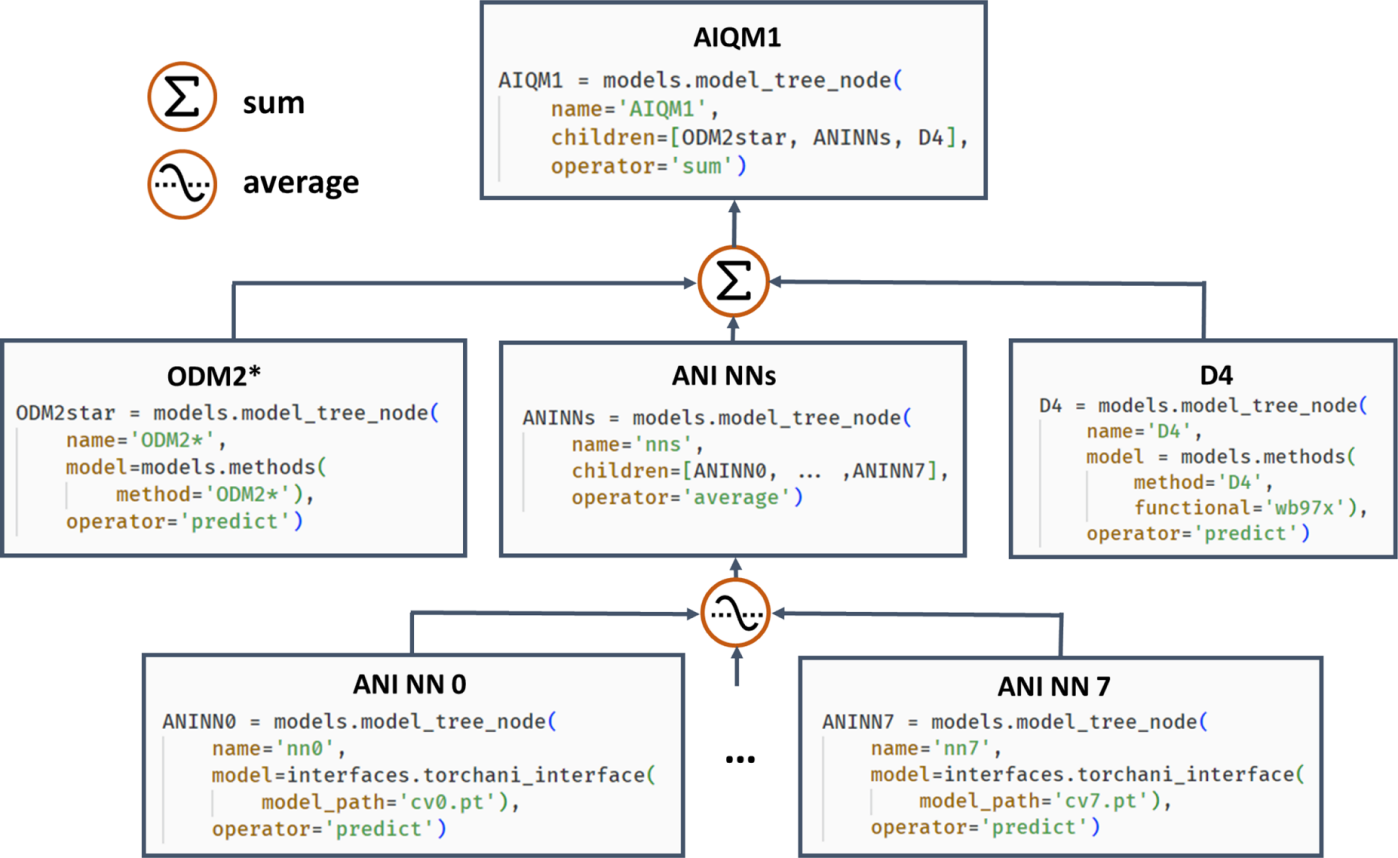
Composite models can be constructed as a model tree in MLatom. Here, an example is shown for the AIQM1 method where the root parent node comprises 3 children, the semiempirical QM method ODM2*, the NN ensemble, and additional D4 dispersion correction. The NN ensemble in turn is a parent of 8 ANI-type NN children. Predictions of parents are obtained by applying an operation “average” or “sum” to children's predictions. The code snippets are shown, too.

Often, it is beneficial to combine several models. One example of such composite models is based on Δ-learning^23^ where the low-level QM method is used as a baseline, which is corrected by an ML model to approach the accuracy of the target higher-level QM method. Another example is ensemble learning^87^ where multiple ML models are created, and their predictions are averaged during the simulations to obtain more robust results and use in the query-by-committee strategy of active learning.^88^ Both of these concepts can also be combined in more complex workflows as exemplified by the AIQM1 method,^32^ which uses the NN ensemble as a correcting Δ-learning model and the semiempirical QM method as the baseline. To easily implement these workflows, MLatom allows the construction of the composite models as model trees; see an example of AIQM1 in Figure 5.

Other examples of possible composite models are hierarchical ML,^25^ which combines several (correcting) ML models trained on (differences between) QM levels, and self-correction,^26^ when each next ML model corrects the prediction by the previous model.

## 5 Simulations

MLatom supports a range of simulation tasks such as single-point simulations, geometry optimizations, frequency and thermochemistry calculations, molecular and quantum dynamics, one- and two-photon absorption, and (ro)vibrational spectra simulations (Figure 1). Most of them need any model that can provide energies and energy derivatives (gradients and Hessians).

### 5.1 Single-Point Calculations

Single-point calculations are calculations of quantum mechanical properties—mainly energies and energy gradients, but also Hessians, charges, dipole moments, *etc*.—for a single geometry. These calculations are very common in ML research in computational chemistry as they are used both to generate the reference data with QM methods for training and validating ML and to make inferences with ML to validate the trained model and generate required data for new geometries. MLatom is a convenient tool to perform single-point calculations not just for a single geometry, as in many QM packages, but for data sets with many geometries.

### 5.2 Geometry Optimizations

**Figure 6:**
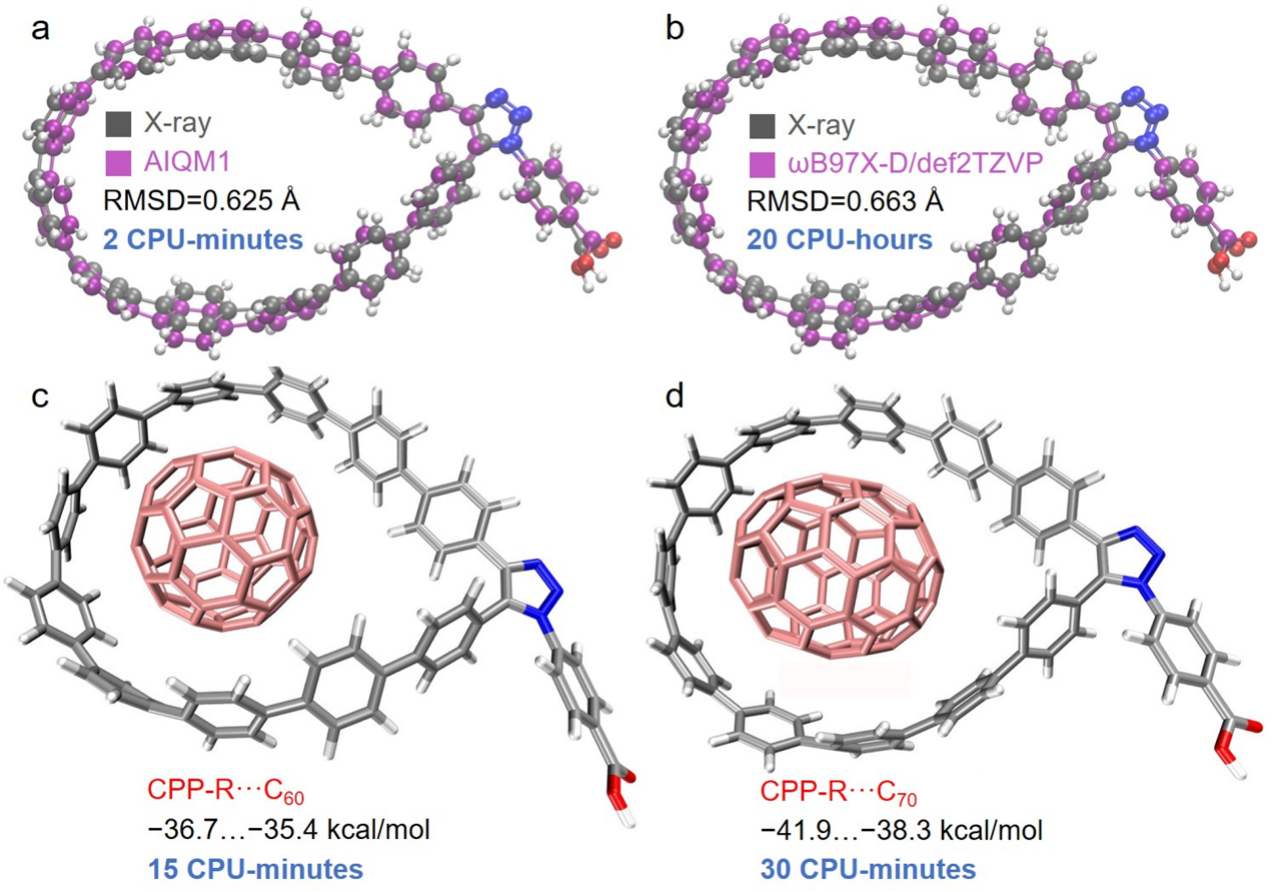
X-ray structure of the functionalized cycloparaphenylene (CPP) nanolasso superimposed with the structure optimized in vacuum at (a) AIQM1 and (b) ωB97X-D/def2-TZVP. Complexes of functionalized CPP and (c) C_60_ and (d) C_70_ with binding energies in kcal/mol calculated at AIQM1 in vacuum. The CPU time for these calculations is also reported.

Locating stationary points on the PES, such as energy minima and transition states, is crucial for understanding the molecular structure and reactivity. Hence, geometry optimizations are among the most important and frequent tasks in computational chemistry. MLatom can locate energy minima and transition states (TS) with any model providing energies and gradients. An example of geometry optimization is given in Figure 2. A practical application of MLatom for efficient and accurate geometry optimization was performed previously for rather large cycloparaphenylene (CPP) nanolassos and their complexes with fullerene molecules (systems with up to 200 atoms, Figure 6).^89^ The AIQM1 method can provide an optimized functionalized CPP structure, which has better agreement with the X-ray structure than that obtained from the DFT method at a speed 600 times faster than the DFT method. In our laboratories, we also use the AIQM1 method to optimize systems with more than a thousand of atoms on a single CPU, while for more computationally intensive tasks such as dynamics of large systems, one can use the pretrained ANI methods. Hessians are also required for the Berny TS optimization algorithm. Once the TS is located, the user can follow the intrinsic reaction coordinate (IRC)^39^ to check its nature. Geometry optimizations can be performed with many algorithms provided by the interfaces to SciPy,^79^ ASE,^18^ or Gaussian.^48^ TS search can be performed with the dimer method^90^ in ASE and the Berny algorithm^91^ in Gaussian. IRC calculations can only be performed with the interface to Gaussian.

The seamless integration of the variety of QM and ML methods for performing geometry optimizations is advantageous because it allows the use of methods from interfaced programs that do not implement some of these simulation tasks by themselves. For example, MLatom can be used to perform TS search with the GFN2-xTB method via an interface to the xtb program, while there is no option for TS search with the latter program. Similarly, Sparrow, which provides access to many semiempirical methods, can only be used for single-point calculations. Since analytical gradients and Hessians are not available for many models and implementations, MLatom also implements a finite-difference numerical differentiation, further expanding the applicability of the models for geometry optimizations.

### 5.3 Frequency Calculations

Simulation of vibrational frequencies is another common and important task in computational chemistry as it is useful to additionally verify the nature of stationary points, visualize molecular vibrations, calculate zero-point vibrational energy (ZPE) and thermochemical properties, and obtain spectroscopic information, which can be compared to experimental vibrational spectra. These calculations can be performed within the ridge-rotor harmonic approximation via an adapted TorchANI implementation^2^ and Gaussian^48^ interface. The latter also allows the calculation of anharmonic frequencies using the second-order perturbative approach.^92^

Similarly to geometry optimizations, MLatom can perform these simulations with any model—ML and QM or their combination—that provides energies. Calculations also need Hessian, and wherever available, analytical Hessian is used. If it is unavailable, semianalytical (with analytical gradients) or fully numerical Hessian can be calculated.

### 5.4 Relative Energy Calculations

**Figure 7:**
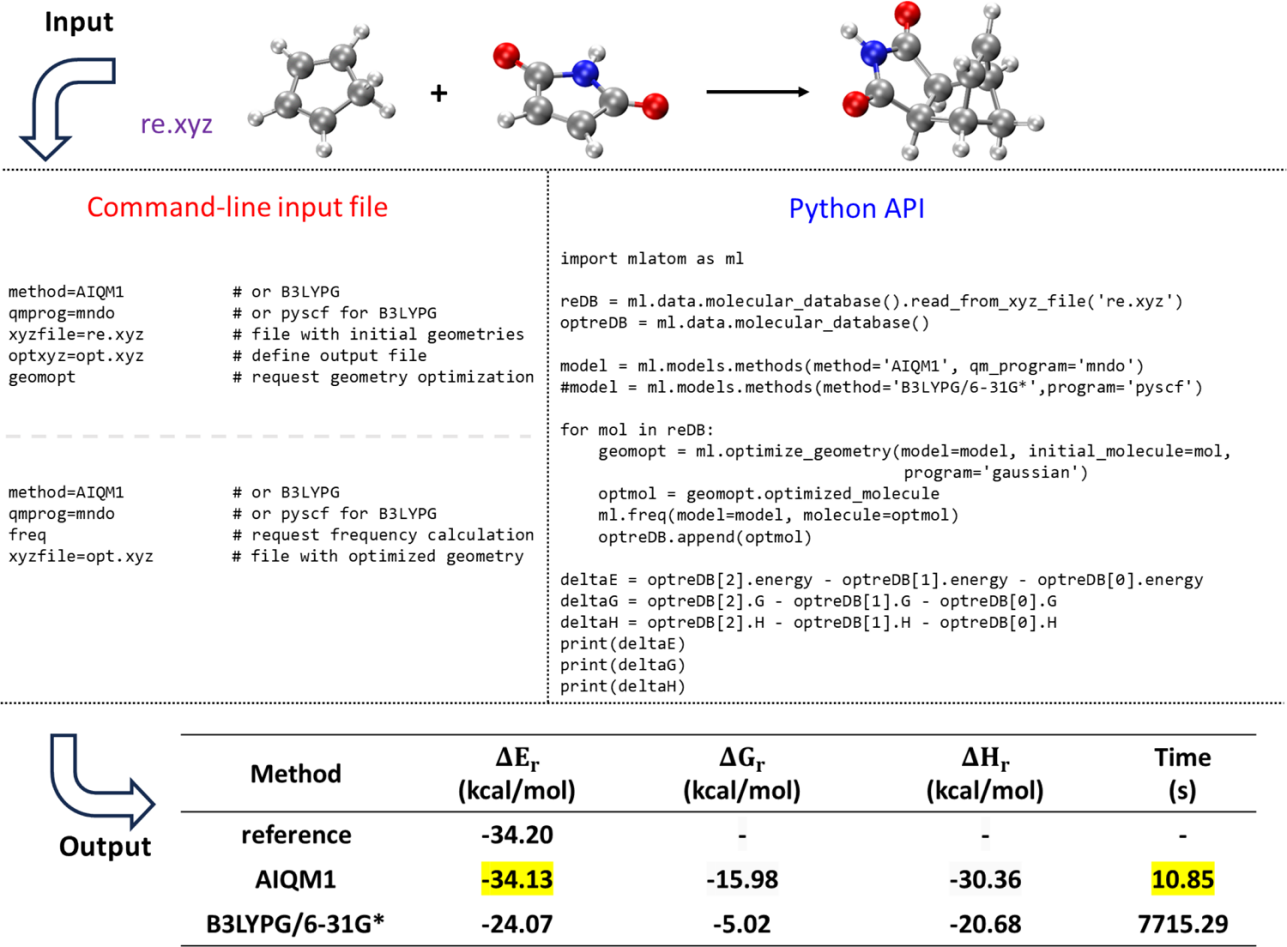
Calculations of ZPVE-exclusive energy, Gibbs free energy, and enthalpy changes in the Diels–Alder reaction of cyclopentadiene and maleimide forming the corresponding endo product with AIQM1 and B3LYPG/6-31G* (from the interface to PySCF; “*G*” in B3LYPG means that we use the B3LYP variant according to the Gaussian program convention). The reference reaction energy is from the GMTKN55 set.^93^

Relative energy is crucial for understanding and predicting various aspects of chemical behavior, from kinetics to thermodynamics, e.g., via calculating reaction energies, barrier heights, isomerization energies, and molecular stabilities. MLatom can produce various types of energies for molecules such as ZPE-exclusive and inclusive total energies, enthalpies, entropies, Gibbs free energies, and internal energies. Hence, the package can readily be used to evaluate different types of relative energies, e.g., the reaction enthalpies and Gibbs free energies as shown for investigating which fullerene molecules bind stronger to the cycloparaphenylene nanolassos (Figure 6) and for the Diels–Alder reaction of cyclopentadiene and maleimide (Figure 7).

#### 5.4.1 Calculation of Heats of Formation

The special type of relative energy calculation is evaluation of heats (enthalpies) of formation. MLatom uses the scheme analogous to those employed in the *ab initio*(94) and semiempirical QM calculations^49^ to derive heats of formation:
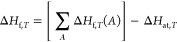
2where Δ*H*_f,*T*_(*A*) is the experimental enthalpies of formation of the free atom *A* and Δ*H*_at,*T*_ is the atomization enthalpy. In AIQM1 and ANI-1ccx, we use the same Δ*H*_f,*T*_(*A*) values as other semiempirical QM methods, i.e., 52.102, 170.89, 113.00, and 59.559 kcal/mol for elements H, C, N, and O, respectively.^50^

The atomization enthalpy Δ*H*_at,*T*_ can be obtained from the difference between molecular *H*_*T*_ and atomic absolute enthalpies *H*_*T*_(*A*):
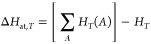
3

Analogous to *ab initio* methods, harmonic-oscillator and rigid-rotor approximations are explicitly considered in the calculation of absolute enthalpies:
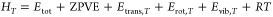
4
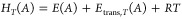
5where *E*_tot_ and *E*(*A*) are the total energy of the molecule and free atom, respectively, and ZPVE is the zero-point vibrational energy. *E*_trans,*T*_, *E*_rot,*T*_, and *E*_vib,*T*_ are the translational, rotational, and vibrational thermal contributions, respectively, and *R* is the gas constant.

The scheme requires knowledge of the free atom energies *E*(*A*). Any model able to calculate them can be used for predicting heats of formation. This is straightforward for QM methods and also possible for ML models if the energies of isolated atoms were included in the training data. However, if the ML-based models are trained only on molecular species, as is commonly done, they cannot be expected to produce reasonable heats of formation. In the case of the pretrained models supported by MLatom, we have previously fitted free atom energies (see Table 3) for AIQM1 and ANI-1ccx methods to reproduce experimental heats of formation for a set of common molecules because the NNs in these methods were not trained on an isolated atom.^32,36^ As a result, both methods can provide heats of formation close to chemical accuracy with speed orders of magnitude higher than those of alternative, high-accuracy QM methods. In addition, we provide an uncertainty quantification scheme based on the deviation of NN predictions in these methods to tell the users when the predictions are confident. This was useful to find errors in the experimental data set of heats of formation.^36^

**Table 3:** Atomic Energies (in hartree) of AIQM1 and ANI-1ccx Used in Heat of Formation Calculations^32,36^

element	AIQM1	ANI-1ccx
H	–0.50088038	–0.50088088
C	–37.79221710	–37.79199048
N	–54.53360298	–54.53379230
O	–75.00986203	–75.00968205

**Figure 8:**
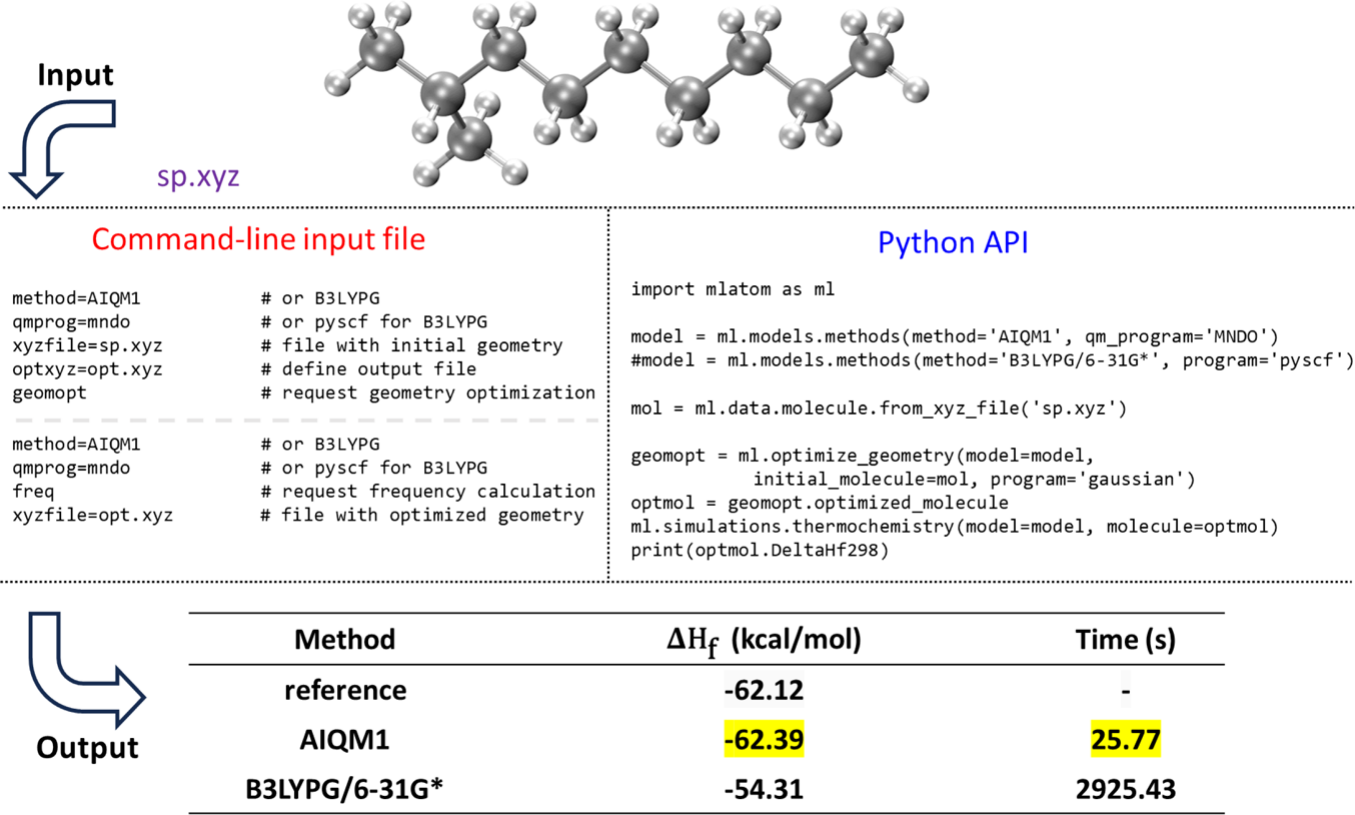
Calculation of heats of formation of 2-methylnonane with AIQM1 and B3LYPG/6-31G* (from the interface to PySCF; “*G*” in B3LYPG means that we use the B3LYP variant according to the Gaussian program convention) compared to the experiment.^95^

An example of using MLatom to calculate the heats of formation with the AIQM1 and B3LYP/6-31G* methods is shown in Figure 8. AIQM1 is both faster and more accurate than B3LYP, as can be seen by comparing the values with the experiment. This is also consistent with our previous benchmark.^36^

### 5.5 Molecular Dynamics

**Figure 9:**
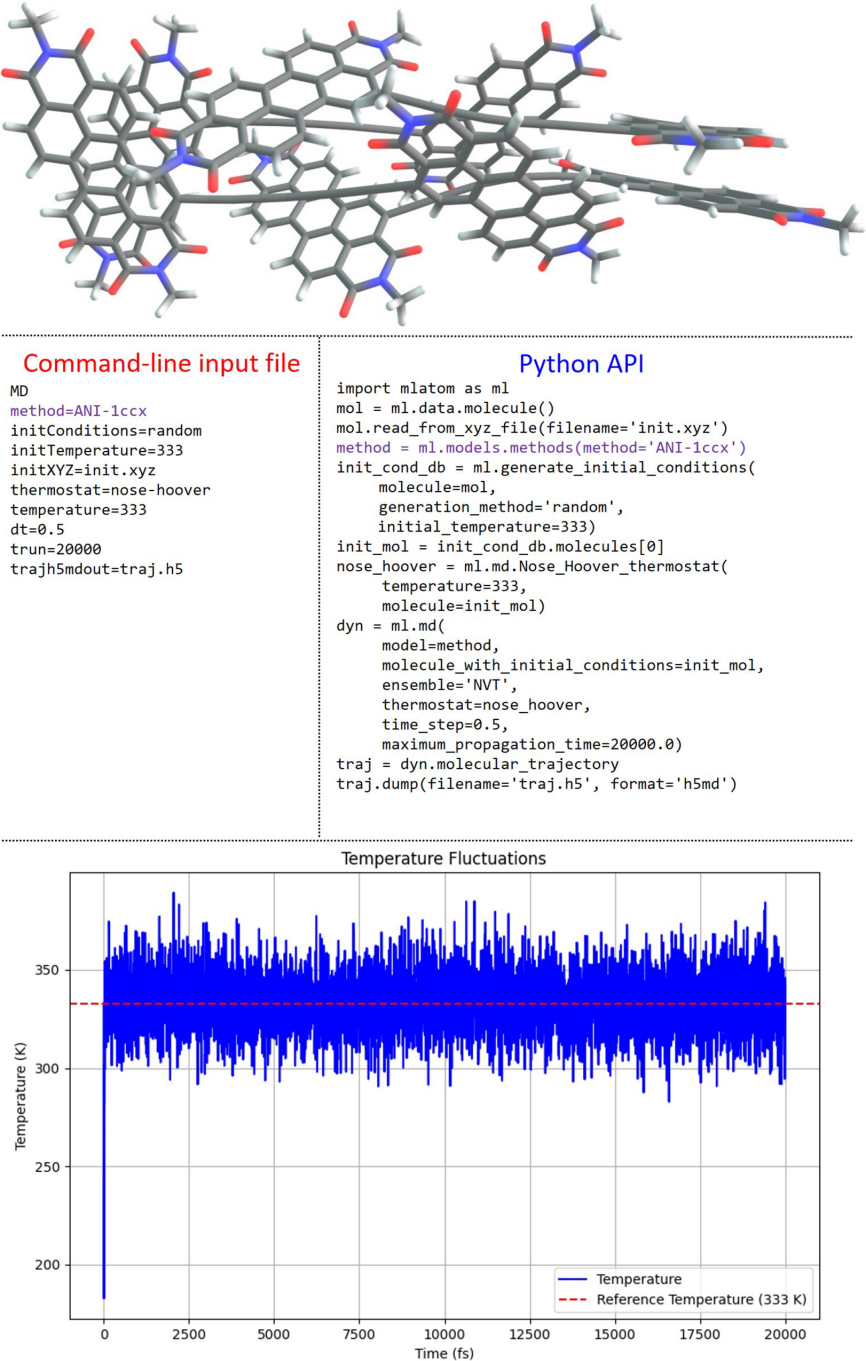
Structure of the (POP)_4_ complex,^98^ a quadruple assembly of octatetrayne-bridged *ortho*-perylene diimide dyads. The command-line input file and the Python script used for NVT MD propagation with the ANI-1ccx method for this molecule are provided. The evolution of the temperature over time during NVT MD is also shown.

**Figure 10:**
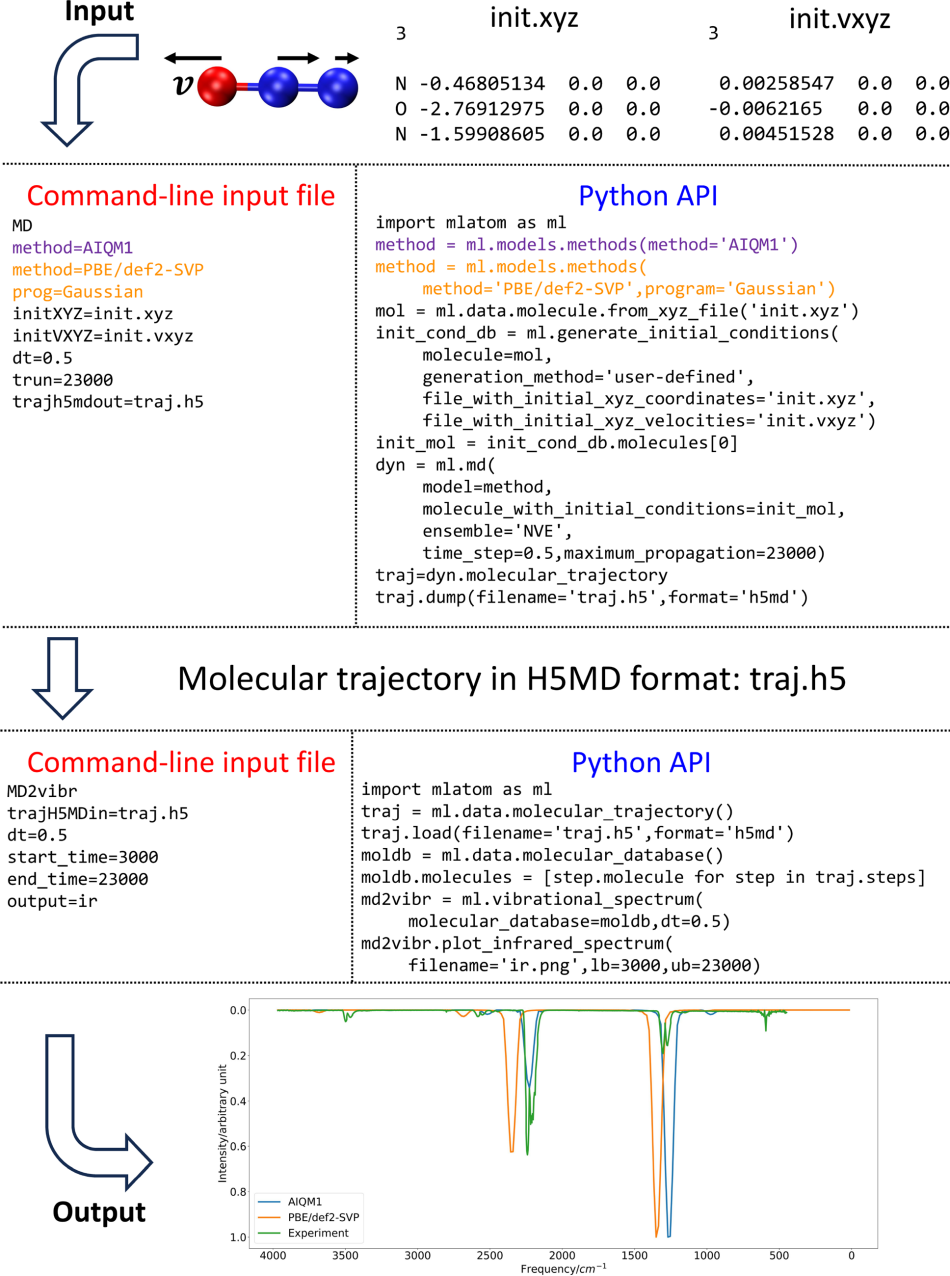
Propagation of MD with AIQM1 and PBE/def2-SVP (from the interface to Gaussian) and the IR spectra of the N_2_O molecule derived from trajectories. MLatom generates spectra for each method; here, the results are collated and shown together with the experimental spectrum^100^ for comparison.

Molecular dynamics propagates nuclear motion based on the equation of motion according to the classical mechanics.^96^ This requires knowledge of forces acting on nuclei, which are typically derived as the negative of the potential energy gradients (i.e., negative of the derivatives of the model for potential energies) for conservative forces. Due to the high cost of the approach, it is most commonly used with molecular mechanics force fields,^97^ but often, calculations based on QM methods are possible in variants called *ab initio* or Born–Oppenheimer MD (BOMD).^96^ The proliferation of ML potentials makes it possible to perform BOMD-quality dynamics at a cost comparable to molecular mechanics force fields or much faster than commonly used DFT-based BOMD,^40−44^ which allows routine simulations of large systems such as a quadruple assembly of octatetrayne-bridged *ortho*-perylene diimide dyads with ca. 400 atoms^98^ at ANI-1ccx (Figure 9). The accuracy of such simulations can be also high; for example, the IR spectra obtained from the MD with AIQM1 method are more accurate than those from a much slower DFT MD (Figure 10).^99^

MLatom has a native implementation of MD supporting any kind of model that provides forces, not necessarily conservative.^99^ Currently, simulations in NVE and NVT ensembles,^101^ based on the velocity Verlet algorithm,^102^ are possible. NVT simulations can be carried out with the Andersen^101,103^ and Nosé–Hoover^104,105^ thermostats, and the implementation of other thermostats is expected to be available in the future. Trajectories can be saved in different formats, including plain text, JSON, and more compact H5MD^29^ database formats. The Nosé–Hoover thermostat is a deterministic thermostat that couples the system to a thermal bath through extra terms in the Hamiltonian. Its theory and implementation details are described elsewhere.^99^ Here, we briefly mention the relevant methodology^101,103^ used in the Andersen thermostat. In this thermostat, the system is coupled to a heat bath by stochastically changing the velocity of each atom. The changing frequency (or collision frequency) is controlled by the tunable parameter *v*. The collisions follow the Poisson distribution, so that the probability of changing the velocity of each atom during a time step Δ*t* is *v*Δ*t*. If the atoms collide, new velocities will be assigned to them, sampled from a Maxwell–Boltzmann distribution at target temperature *T*.

Multiple independent MD trajectories can be propagated in parallel, dramatically speeding up the calculations. In addition, we made an effort to better integrate the KREG model implemented in Fortran into the main Python-based MLatom code, which makes MD with KREG very efficient.

Note that MD can also be propagated without forces using the concept of the 4D-spacetime AI atomistic models, which directly predict nuclear configurations as a function of time.^85^ Our realization of this concept, called the GICnet model, is currently available in a publicly available development version of MLatom version.^85^

The above implementations can propagate MD on an adiabatic potential energy surface, i.e., typically for ground-state dynamics. Nonadiabatic MD based on the trajectory surface hopping algorithms can also be performed with the help of MLatom, currently, via Newton-X's^106^ interface to MLatom.^27,107,108^ MLatom also supports quantum dissipative dynamics, as described in the next section.

### 5.6 Quantum Dissipative Dynamics

It is often necessary and beneficial to treat the entire system quantum mechanically and also include the environmental effects.^109^ This is possible via many quantum dissipative dynamics (QD) algorithms, and an increasing number of ML techniques were suggested to accelerate such simulations.^107^ MLatom allows performing several unique ML-accelerated QD simulations using either a recursive scheme based on KRR^110^ or a conceptually different AI-QD approach^38^ predicting the trajectories as a function of time or the OSTL technique^111^ outputting the entire trajectories in one shot. These approaches are enabled via an interface to a specialized program MLQD.^112^

In the recursive KRR scheme, a KRR model is trained, establishing a map between the future and past dynamics. This KRR model, when provided with a brief snapshot of the current dynamics, can be leveraged to forecast future dynamics. In the AI-QD approach, a convolution neural network (CNN) model is trained mapping simulation parameters and time to the corresponding system’s state. Using the trained CNN model, the state of the system can be predicted at any time without the need to explicitly simulate the dynamics. Similarly, the ultrafast OSTL method utilizes a CNN-based architecture and, based on simulation parameters, predicts future dynamics of the system’s state up to a predefined time in a single shot. In addition, as optimization is a key component in training, users can optimize both KRR and CNN models using MLatom’s grid search functionality for KRR and Bayesian optimization via the hyperopt^77^ library for CNN. Moreover, we also incorporate the autoplotting functionality, where the predicted dynamics is plotted against the provided reference trajectory.

### 5.7 Rovibrational (Infrared and Power) Spectra

Rovibrational spectra can be calculated in several ways with MLatom. The simplest method is by performing frequency calculations on an optimized molecular geometry. This requires any model providing Hessians and, preferably, dipole moments. Another one is performing molecular dynamics simulations with any model providing energy gradients and, then, postprocessing the trajectories.

Both frequency calculations and the MD-based approach require the model to also provide dipole moments to calculate the absorption intensities. If no dipole moments are provided, only frequencies are available, or, in the case of MD, only power spectra rather than IR can be obtained. The IR spectra are obtained via the fast Fourier transform using the autocorrelation function of dipole moment^113,114^ with our own implementation.^99^ The power spectra only need the fast Fourier transform,^113^ which is also implemented^85^ in MLatom.

We have previously shown^99^ that the high quality of the AIQM1 method results in rather accurate IR spectra obtained from MD simulations compared to spectra obtained with a representative DFT (which is also substantially slower; see example in Figure 10) or a semiempirical QM method.

### 5.8 One-Photon UV/Vis Absorption Spectra

**Figure 11:**
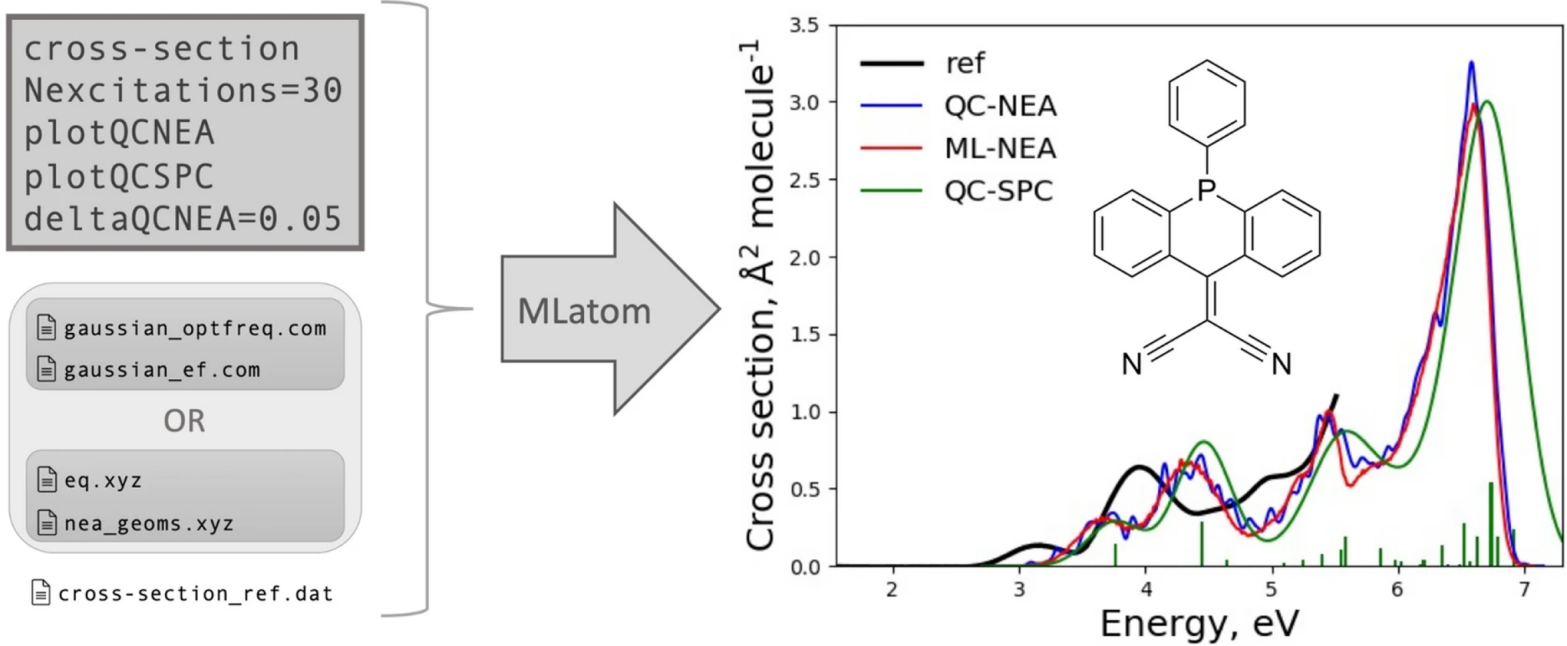
Using MLatom to predict the UV/vis absorption spectra of the acridophosphine derivative molecule with the ML-NEA^30^ method. The MLatom input file and the list of additional required files are shown on the left. The cross section predicted by ML-NEA shown on the right is compared to traditional QC-NEA and the single-point convolution approach (QC-SPC). This figure is adapted from ref (29). Copyright 2021, the Authors.

UV/vis absorption spectra simulations are computationally intensive because they require calculation of excited-state properties. In addition, better-quality spectra can be obtained via the nuclear ensemble approach (NEA),^115^ which necessitates the calculation of excited-state properties for thousands of geometries for high precision. MLatom implements an interpolation ML-NEA scheme^30^ that improves the precision of the spectra with a fraction of the computational cost of traditional NEA simulations (Figure 11). Currently, the ML-NEA calculations are based on interfaces to Newton-X^106^ and Gaussian^48^ and utilize the sampling of geometries from a harmonic Wigner distribution.^116^ This scheme also automatically determines the optimal number of required reference calculations, providing a user-friendly, black-box implementation of the algorithm.^29^

### 5.9 Two-Photon Absorption

**Figure 12:**
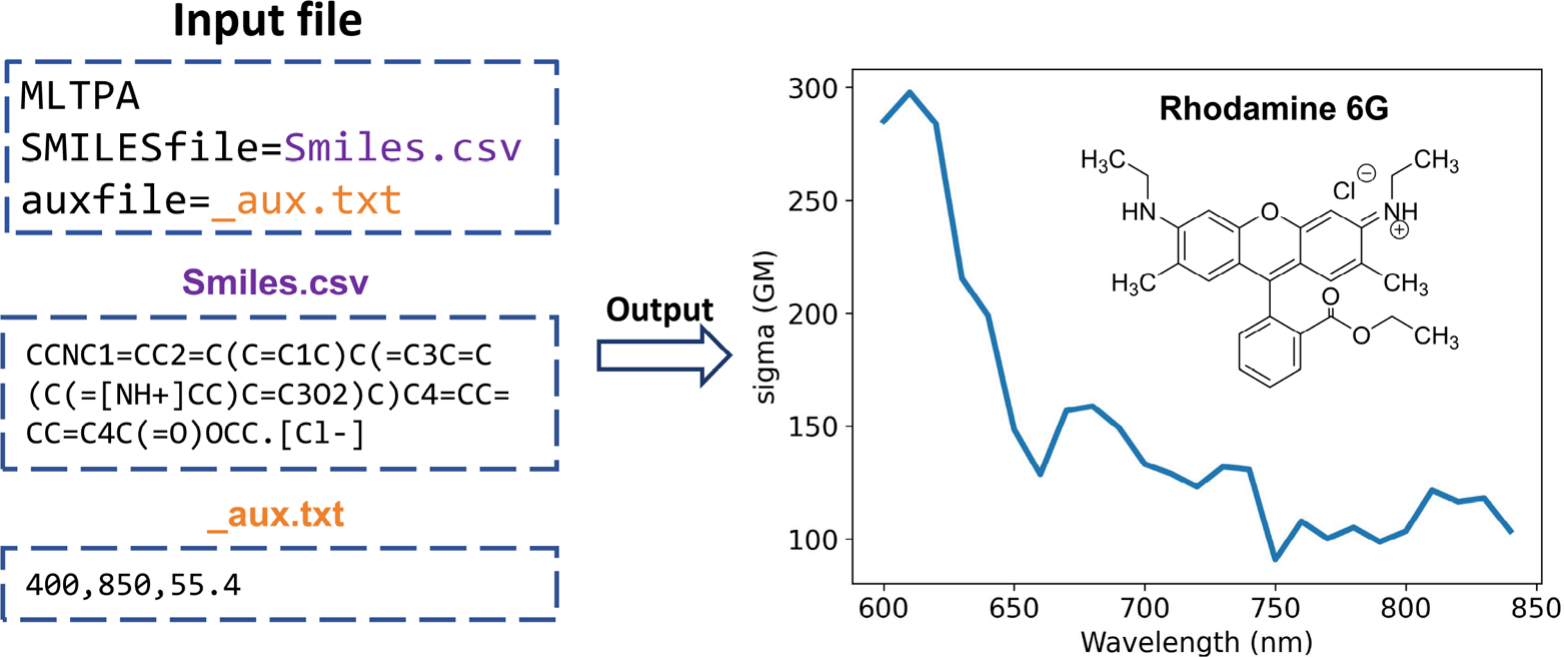
Using MLatom to predict the two-photon absorption cross section of the Rhodamine 6G molecule with the ML-TPA approach. The MLatom command-line input file and additional files are shown on the left. The cross section predicted by ML-TPA is shown on the right.

Beyond one-photon absorption, MLatom has an implementation of a unique ML approach for calculating two-photon absorption (TPA) cross sections of molecules just based on their SMILES strings,^45^ which are converted into the required descriptors using the interface to RDKit,^117^ and solvent information.^31^ This ML-TPA approach is very fast with accuracy comparable to that of much more computationally intensive QM methods. We provide an ML model pretrained on experimental data. ML-TPA was tested in real laboratory settings and was shown to provide a good estimate for new molecules not present in the training experimental database. An example of using ML-TPA to predict two-photon absorption is shown in Figure 12.

## 6 Machine Learning

In Sections 4 and 5, we discussed the supported types of models and how they can be applied to simulations. Here, we briefly overview the general considerations for training and validating the ML models with MLatom. The models share the standard MLatom’s conventions for input, output, training, hyperparameter optimization, and testing, which allows one to conveniently switch from one model to another and benchmark them.

### 6.1 Training

To create an ML model, the user has to choose and train the ML model and prepare data. MLatom provides many tools for the different stages of this process. The model can be either chosen from a selection of provided types of ML models with a predefined architecture or customized based on available algorithms and preset models. Once a model is chosen, it must be trained, and, in many cases, it is advisable or even required (particularly in the case of the kernel methods) to optimize its hyperparameters, which can be done as explained in Section 6.2.

For training, the data set should be appropriately prepared. MLatom has strict naming conventions for data set splits to avoid any confusion when changing and comparing different model types. All of the data that are used directly or indirectly for creating an ML model are called the training set. This means that the validation set, which can be used for hyperparameter optimization or early stopping during NN training, is a subset of the training set. Thus, the part of the training set remaining after excluding the validation set is called the subtraining set and is actually used for training the model, i.e., optimizing model parameters (weights in NN terminology and regression coefficients in kernel method terminology).

MLatom can split the training data set into the subtraining and validation data subsets or create a collection of these subsets via cross-validation.^24,29^ The sampling into the subsets can be performed randomly or using furthest-point or structure-based sampling.

In the case of kernel methods, the final model in MLatom is typically trained on the entire training set after hyperparameter optimization. This is possible because the kernel methods have a closed analytical solution to finding their regression coefficients, and after hyperparameters are appropriately chosen, overfitting can be mitigated to a great extent. In the case of NNs, the final model is the one trained on the subtraining set because it would be too dangerous to train on the entire training set without any validation subset to check for the signs of overfitting.

#### 6.1.1 Training Predefined Types of ML Models

**Figure 13:**
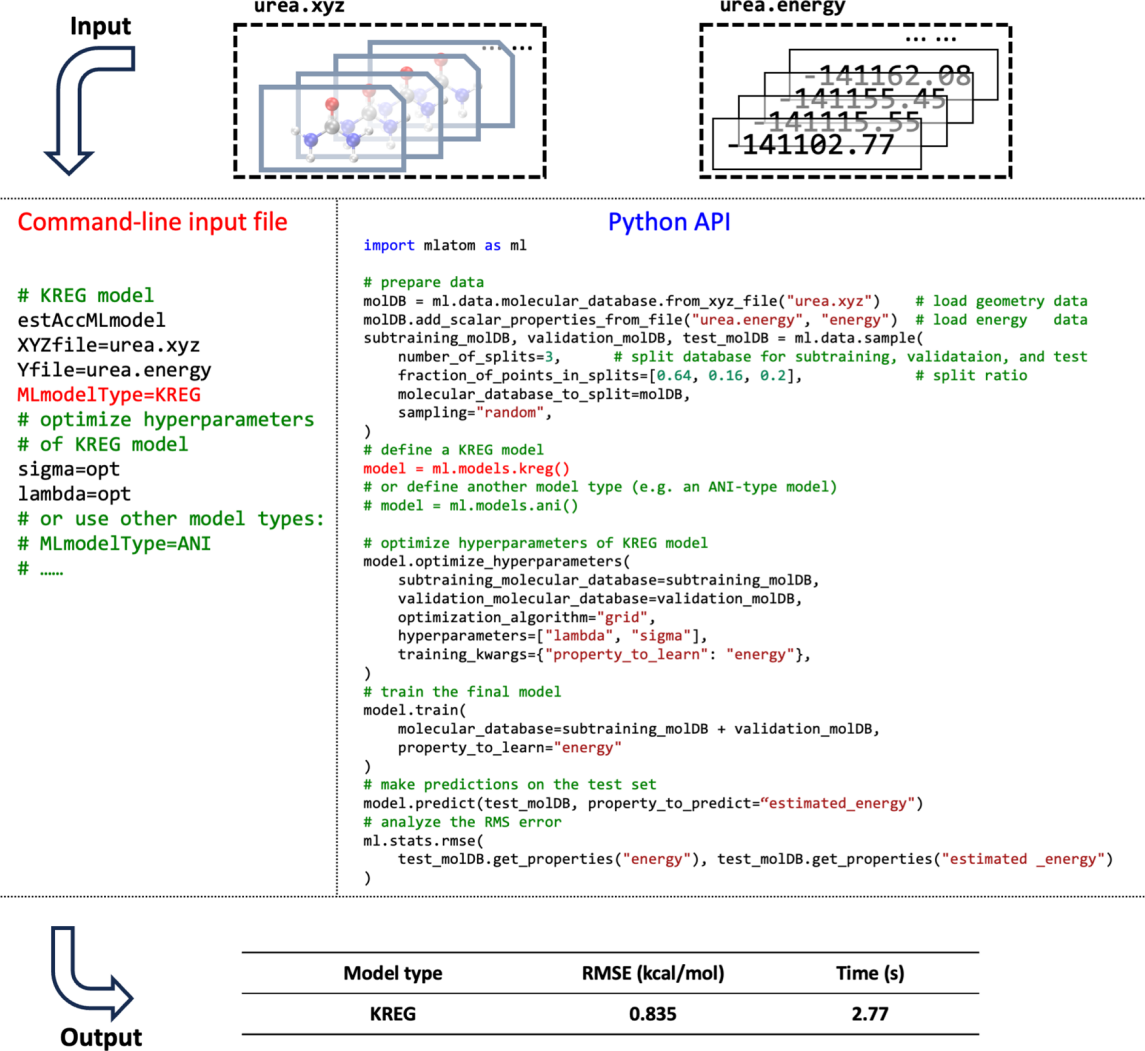
Side-by-side comparison of the usage of MLatom in both the command-line mode and via Python API for training and testing the KREG model on a 1000-point data set on the urea molecular PES data set randomly sampled from the WS22 database.^121^ Hyperparameter optimization of the KREG model required is also shown. Calculations were run on a 36 Intel(R) Xeon(R) Gold 6240 CPU @ 2.60 GHz.

Most predefined types of ML models, such as ANI-type or KREG models, expect *XYZ* molecular coordinates as input. This should be either provided by the user or can be obtained using MLatom’s conversion routines, e.g., from the SMILES strings,^118^ which rely on OpenBabel's^119^ Pybel API. These models have a default set of hyperparameters, but, especially in the case of kernel methods such as KREG, it is still strongly advised to optimize them. The models can be, in principle, trained on any molecular property. Most often, they are used to learn PESs and hence require energy labels in the training set. The PES model accuracy can be greatly improved if the energy gradients are also provided for training. Thus, the increased training time is usually justified.^40,120^ An example of training and testing the KREG model on a data set with energies and energy gradients for the urea molecule in the WS22 database^121^ is shown in Figure 13. The KREG model is both fast to train and accurate (achieved an RMSE below 1 kcal/mol within a few seconds), which is a typical situation for small-size molecular databases, while for larger databases, NN-based models might be preferable.^40^ Command-line and Python script inputs for using a different type of ML model (e.g., ANI-type^2^) are also shown in the figure as comments.

#### 6.1.2 Designing and Training Custom ML Models

MLatom’s user can also create models on any set of input vectors and labels using a variety of KRR kernel functions. In this case, hyperparameter optimization is strongly advised too. In all other aspects, training of such KRR models is similar to training the predefined models, i.e., the preparation of the data set is also performed by splitting it into the required subsets for training and validation.

Importantly, the user can construct models of varying complexity using a model tree implementation. Special cases of such composite models are Δ-learning and self-correcting models, and they can be trained similarly to other ML models by supplying input vectors or *XYZ* coordinates and labels. In the case of Δ-learning, the user must supply the baseline values. For other more complicated models, the user must train and combine each component separately.

### 6.2 Hyperparameter Optimization

The performance of ML models strongly depends on the chosen hyperparameters, such as the regularization parameters for training kernel methods and the number of layers in NNs. Hence, it is often necessary to optimize the hyperparameters to achieve reasonable results and to improve the accuracy. The hyperparameter optimization commonly requires multiple trainings, making it an expensive endeavor, and caution must be paid in balancing performance/cost issues.

MLatom can optimize hyperparameters by minimizing the validation loss using one of the many available algorithms. The validation loss is usually based on the error in the validation set, which can be a single hold-out validation set or a combined cross-validation error.

For a few hyperparameters, the robust grid search on the log or linear scale can be used to find optimal values. It is a common choice for kernel methods (see Figure 13 for an example of optimizing hyperparameters of the KREG model, which is the kernel method). For a larger number of hyperparameters, other algorithms are recommended instead. Popular choices are Bayesian optimization with the tree-structured Parzen estimator (TPE)^78^ and many SciPy optimizers.

The choice of the validation loss also matters. In most cases, MLatom minimizes the root-mean-square error (RMSE) for the labeled data. However, when multiple labels are provided, i.e., energies and energy gradients for learning PES, the choice should be made on how to combine them in the validation loss. By default, MLatom calculates the geometric mean of the RMSEs for energies and gradients.^29^ The users can also choose a weighted sum of RMSEs, but in this case, they must choose the weight. In addition, the user can supply MLatom with any custom validation loss function, which can be arbitrarily complicated.

### 6.3 Evaluating Models

Once the model has been trained, it is common to evaluate its generalization ability before deployment in production simulations. MLatom provides dedicated options for such evaluations. The simplest and one of the most widespread approaches is calculating the error for the independent hold-out test set not used in the training. To emphasize, in MLatom terminology, the test set has no overlap with the training set, which might consist of the subtraining and validation subsets.^29^ Alternatively, cross-validation and its variant leave-one-out cross-validation are recommended whenever computationally affordable, especially for small data sets. MLatom provides a broad range of error measures for the test set, including RMSE, mean absolute error (MAE), mean signed error, the Pearson correlation coefficient, the *R*^2^ value, outliers, *etc*.^29^ The testing can be performed with training and hyperparameter optimization for most models, including Δ-learning and self-correcting models.

Since the errors depend on the size of the training set, the learning curves showing this dependence are very useful for comparing different models.^29^ MLatom can generate the learning curves, which have been instrumental in preparing guidelines for choosing the ML interatomic potential.^40^

## 7 Summary

MLatom 3 is a unique software package combining machine learning and quantum mechanical models for accelerating and improving the accuracy of computational chemistry simulations. It can be used as a black-box package accepting input files with a simple structure or as a transparent Python module enabling custom workflows. MLatom provides access to pretrained models such as AIQM1 and ANI-1ccx aiming at high accuracy of the coupled-cluster level, making them more accurate and much faster than common DFT approaches for ground-state properties of closed-shell organic molecules. Another special pretrained model can be used to simulate two-photon absorption spectra.

The user of MLatom has an option to create their own models. Predefined ML architectures of the MACE, ANI-type, KREG, PhysNet, GAP-SOAP, DPMD, or sGDML make it easier. Alternatively, the custom models of varying complexity and based on combinations of both ML and QM models, such as Δ-learning, can be easily built with the package. MLatom provides a toolset for training, hyperparameter optimization, and performance analysis of the models.

This wide variety of models can be used for single-point calculations on large data sets, geometry optimizations, calculation of rovibrational (frequencies and IR spectra) and thermochemical (enthalpies, entropies, and heats of formation) properties, molecular dynamics, and UV/vis absorption spectra. The ML models can also be trained and used for quantum dissipative dynamics simulations.

For developers, MLatom provides a flexible platform for implementation of the new interfaces as they just need to provide a new class supporting prediction (and optionally training) with the new model. For example, the implementation of MACE was done in one working day, and another working day was needed for testing. Once implemented, these models can be readily used for simulations.

The richness of the MLatom functionality is available open-source and can be exploited on the XACS cloud computing service. We also welcome new contributions to the package. The package is accompanied by extensive and detailed manuals and tutorials that are developed and improved in close connection with teaching computational chemistry and machine learning in regular workshops and university courses.

## Data Availability

The MLatom code is open-source and available both on GitHub (https://github.com/dralgroup/mlatom, main GitHub repository) and PyPI (i.e., it can be installed via the command pip install mlatom). The contributions to the main GitHub repository of MLatom are highly welcome and can be done via pull requests from branches (on request) and forks that the contributors may also create for their private developments of methods and features. The pull requests may be incorporated into official releases after the review and eventual adjustments by the main developers’ team managing the main GitHub repository. The simulations can also be run on the MLatom@XACS cloud computing service on https://XACScloud.com.

## Notes

No data were generated for this article.
